# Genetic Parameter Estimates and Associations Between Clutch Length and Hen-Day Egg Production Traits in Thai Native Chickens Under Heat Stress

**DOI:** 10.3390/ani16040681

**Published:** 2026-02-21

**Authors:** Piriyaporn Sungkhapreecha, Vibuntita Chankitisakul, Wuttigrai Boonkum

**Affiliations:** 1 Department of Animal Science, Faculty of Agricultural Technology, Rajamangala University of Technology Thanyaburi, Pathum Thani 12110, Thailand; piriyaporn_s@rmutt.ac.th; 2 Network Center for Animal Breeding and Omics Research, Khon Kaen University, Khon Kaen 40002, Thailand; vibuch@kku.ac.th; 3 Department of Animal Science, Faculty of Agriculture, Khon Kean University, Khon Kean 40002, Thailand

**Keywords:** heat tolerance, genetic parameter, clutch length, hen-day egg production, slow-growing chicken

## Abstract

Improving egg production under heat stress remains a major challenge in tropical poultry breeding. This study demonstrates that clutch length and hen-day egg production are robust indicators of genetic variation in heat tolerance and directly applicable to climate-resilient breeding programs in Thai native chickens. Records from 2400 Pradu Hang Dum hens across five generations were analyzed using a reaction-norm repeatability model with the temperature–humidity index (THI) as an environmental covariate. A THI threshold of 74 was identified, beyond which heat stress altered the genetic expression of both traits. Heritability estimates declined with an increasing THI, from 0.49 to 0.32 for clutch length and from 0.37 to 0.26 for hen-day egg production, indicating reduced additive genetic control under heat stress. Genetic correlations between baseline performance and heat-stress sensitivity were moderately to strongly negative (−0.46 to −0.54), revealing antagonism between productivity under thermoneutral conditions and heat tolerance. Reaction-norm breeding values showed substantial genotype-by-environment interactions, highlighting heterogeneity in heat-stress responses among genotypes. Despite thermal challenges, positive genetic trends were observed, with an average genetic trend of 1.34 eggs per generation for clutch length and 8.8 percent per generation for hen-day egg production. These results demonstrate that genetic improvement can be sustained under heat stress and support the integration of THI-based reaction-norm evaluations to identify genotypes combining reproductive efficiency with enhanced heat tolerance for climate-resilient breeding programs.

## Simple Summary

Native chickens are widely raised in tropical regions due to their adaptation to local environments; however, egg production remains low and sensitive to heat stress. This study evaluated the effects of heat stress on egg-laying patterns and genetic potential in Thai native chickens, focusing on clutch length and hen-day egg production. Using multi-generational data, both traits declined with increasing temperature and humidity. Variation in responses among chickens indicated genetic differences in heat tolerance, while genetic improvements in egg production remained achievable across generations. These results demonstrate that clutch length and hen-day egg production are effective indicators for identifying heat-tolerant chickens and support their use in sustainable breeding programs for tropical environments.

## 1. Introduction

Poultry production plays an important role in food security and rural livelihoods, particularly in tropical regions where native chicken breeds remain integral to smallholder production systems [1,2,3,4,5,6,7]. Native chickens are widely raised under backyard and semi-intensive conditions and valued for their adaptability, meat quality, and cultural significance [8,9]. These production systems rely heavily on reproductive efficiency to ensure household-level food availability and income stability, making fertility-related traits in native chickens directly relevant to food security and rural livelihoods. However, despite these advantages, the egg production of native chickens (with an average annual egg production of between 60 to 195 eggs/hen) remains substantially lower than that of commercial layer strains (with an average annual egg production between 280 to 330 eggs/hen) [10,11,12,13], characterized by short laying persistency, variable clutch lengths, and low hen-day egg production [12,14,15]. Improving egg production efficiency in native chickens therefore remains a key challenge for sustainable breeding programs.

This challenge is further intensified by heat stress associated with hot and humid tropical climates. Elevated ambient temperature and humidity disrupt reproductive physiology, reduce feed intake, and impair hormonal regulation, leading to marked declines in egg production [16,17,18]. In Thailand, daily maximum temperatures frequently exceed the thermoneutral zone for poultry, resulting in temperature–humidity index (THI) values that negatively affect laying performance [19]. Although native chickens are generally more heat tolerant than commercial layers, substantial variation in productivity across seasons suggests that environmental sensitivity remains a critical constraint [19,20,21,22]. Importantly, such environmental pressures do not affect all birds equally, indicating underlying genetic variation in heat-stress responses.

Clutch length reflects the number of consecutive laying days and directly captures the stability of ovarian function and endocrine regulation underlying egg production. In contrast, cumulative egg production metrics integrate multiple biological and environmental components, including age when the first egg is laid, duration of the recording period, and management interventions, which can obscure environmentally driven variation in reproductive continuity. As a result, clutch length is more sensitive to changes in environmental conditions and better suited to detecting genotype-by-environment (G × E) interactions that influence laying persistency rather than total output [12,23,24]. In commercial poultry, a greater clutch length is strongly associated with higher hen-day egg production and has been proposed to be an effective indirect selection criterion [12]. However, in native chickens, the genetic architecture of clutch length—particularly its response to heat stress and its genetic relationship with egg production—remains poorly understood. Evidence from other avian species, such as Reeves’s pheasant [25] and the chestnut-crowned babbler [26], suggests that clutch traits are sensitive to climatic conditions, yet the magnitude of G × E interactions and their implications for selection under heat stress have not been adequately quantified in native chicken populations.

Recent advances in quantitative genetics emphasize the need to explicitly account for environmental gradients in genetic evaluations. Reaction-norm models, which incorporate environmental variables such as the temperature–humidity index (THI) as covariates, allow estimation of genetic parameters across gradients of environmental stress in several livestock, such as pig, cattle, and chickens [27,28,29]. These models facilitate identification of genotypes with superior resilience under fluctuating conditions. In cattle and pigs, THI-based reaction-norm models have proven valuable for breeding heat-tolerant animals [30,31]. In poultry, applying similar models has revealed genetic variability in heat tolerance, supporting their use in designing robust selection indices [13,19]. However, such approaches have not yet been widely implemented in Thai native chicken breeding. Incorporating the THI into genetic evaluations of clutch length and hen-day egg production could provide critical insights into genotype-by-environment (G × E) interactions and enable the development of selection strategies that simultaneously improve productivity and resilience. Therefore, this study aimed to investigate the genetic and phenotypic responses of clutch length and hen-day egg production to heat stress using THI-based reaction-norm models and evaluate their potential as indicator traits for selecting chickens that combine reproductive efficiency with enhanced heat tolerance.

## 2. Materials and Methods

### 2.1. Animal Ethics and Animal Management

This study was conducted using Thai native chickens (Pradu Hang Dum) maintained at the Network Center for Animal Breeding and Omics Research, Faculty of Agriculture, Khon Kaen University, Thailand. In total, 94,752 clutch length and hen-day egg production records from 2400 hens in a closed-nucleus breeding population across five consecutive generations (G1–G5) were analyzed. All birds were raised under open-sided housing systems with natural ventilation and exposed to ambient environmental conditions. At 20 weeks of age, the laying hens were moved from floor pens bedded with rice husks to individual battery cages (20 × 45 × 40 cm), where they were housed throughout the laying period. All birds were fed a standard commercial layer diet at 110 g/day (containing 19% crude protein and 2900 kcal ME/kg) and had ad libitum access to water. This feeding regimen was consistent across all generations, and it was maintained until the end of the experiment at 40 weeks of laying age. The lighting program provided 12 h of light per day. Standard vaccination and healthcare protocols were implemented in accordance with the regulations of the Thai Department of Livestock Development. All experimental procedures were approved by the Institutional Animal Care and Use Committee of Khon Kaen University in accordance with the Ethics of Animal Experimentation guidelines set by the National Research Council of Thailand (Approval No. IACUC-KKU-108/68).

### 2.2. Air Temperature and Relative Humidity Data

Weather data were recorded using an ELITECH GSP-6 Temperature Data Logger (Elitech Technology, Inc., Milpitas, CA, USA) installed at the center of the chicken house. Ambient air temperature (Temp) and relative humidity (RH) were measured at 3 h intervals using sensors with manufacturer-specified accuracies of ±0.5 °C and ±3%, respectively. The data logger was verified for proper functionality prior to data collection. Records were visually inspected, and any missing observations were excluded before calculation of the temperature–humidity index (THI) according to the formula proposed by NOAA [32]:

THI = (1.8 × Temp + 32) − (0.55 − 0.0055 × RH) × (1.8 × Temp − 26),

where Temp is the average air temperature in degrees Celsius, and RH is the average relative humidity expressed as a percentage.

### 2.3. Estimation of Genetic Parameters

All data were verified before genetic analysis using the PROC UNIVARIATE procedure in SAS version 9.0 [33] to examine data distribution, including assessments of normality, homogeneity of variance, and outlier detection. Records exceeding ±3 standard deviations were considered outliers and excluded from the analysis (948 records; <1% of total observations). The relationships between clutch length and hen-day egg production throughout the laying period, as well as across temperature–humidity index values, in Thai native chickens were analyzed using Microsoft Excel 2021 for Windows. The effects of THI on the decline in clutch length and hen-day egg production were evaluated using regression analysis via PROC REG in SAS software version 9.0.

The repeatability test-day model using the reaction-norm procedure proposed by Boonkum et al. [27] was employed to estimate the threshold point of heat stress and genetic parameters. Different THI thresholds, from 72 to 82, were tested in the model. These THI threshold ranges were tested because this interval is widely recognized as representing the transition from thermoneutral to moderate–severe heat stress in poultry. Previous studies have reported that physiological and productive responses begin to deteriorate when THI exceeds approximately 72, with progressively greater impacts observed above 78–80. Therefore, these ranges were selected to capture the biologically relevant onset and progression of heat stress under tropical conditions. A THI function was created to estimate the decline in clutch length and hen-day egg production under heat stress. The repeatability test-day model, THI function, and variance-covariance structure matrix used in this study are defined below.

The repeatability test-day model used is

$$y_{ijkl}={CG}_{i}+{Age}_{j}+{THI}_{k}[f\left(THI\right)]+a_{0l}+a_{1l}[f\left(THI\right)]+p_{0l}+p_{1l}[f\left(THI\right)]+e_{ijkl}$$

where $y_{ijkl}$ is the observation value of clutch length and hen-day egg production for chicken l in the contemporary group between hatch and generation (CG) class i, age (Age) class j, and THI (THI) class k; ${CG}_{i}$ is the fixed effect of the contemporary group (hatch and generation) i; ${Age}_{j}$ is the fixed effect of chicken age j; ${THI}_{k}$ is the fixed effect of chicken THI value k, which is nested with the THI function ([$f\left(THI\right)$]) to describe the changes in clutch length and hen-day egg production according to the change in THI values; $a_{0l}$ is the random additive genetic effect without consideration of heat stress; $a_{1l}$ is the random additive genetic effect for heat stress; $p_{0l}$ is the random permanent environmental effect without consideration of heat stress; $p_{1l}$ is the random permanent environmental effect of heat stress; $e_{ijkl}$ is the random residual effect; and $f\left(THI\right)$ is a function of the THI.

The THI function used is

$$f\left(THI\right)=\left\{\begin{array}{cc} 0, & THI\leq {THI}_{threshold}\;(no\;heat\;stress)\\ THI-{THI}_{threshold}, & THI>{THI}_{threshold}\;(heat\;stress) \end{array}\right.$$

The best fitting degree of the THI was considered based on two statistical criteria: the lowest minus twice the logarithm of the likelihood (−2logL), Akaike’s information criterion (AIC), and Bayesian information criterion (BIC).

The variance–covariance structure used is

$$Var\left[\begin{array}{c} a_{0}\\ a_{1}\\ p_{0}\\ p_{1}\\ e \end{array}\right]=\left[\begin{array}{ccccc} A\sigma_{a0}^{2} & A\sigma_{a01} & 0 & 0 & 0\\ A\sigma_{01} & A\sigma_{a1}^{2} & 0 & 0 & 0\\ 0 & 0 & I\sigma_{p0}^{2} & I\sigma_{p01} & 0\\ 0 & 0 & I\sigma_{p01} & I\sigma_{p1}^{2} & 0\\ 0 & 0 & 0 & 0 & I\sigma_{e}^{2} \end{array}\right]$$

where $\sigma_{a0}^{2}$ is additive genetic variance without consideration of heat stress; $\sigma_{a1}^{2}$ is the additive genetic variance with heat stress; $\sigma_{p0}^{2}$ is the permanent environmental variance without consideration of heat stress; $\sigma_{p1}^{2}$ is the permanent environmental variance with heat stress;${\;\sigma }_{e}^{2}$ is the residual variance; A is the numerator relationship matrix; and I is an identity matrix. The threshold points of heat stress and variance components were estimated with the average information-restricted maximum likelihood algorithm using the BLUPF90+ software package (version 1.70; University of Georgia, Athens, GA, USA) [34]. Initial values for (co)variance components were obtained from preliminary analyses and updated iteratively. Convergence was assumed when the change in log-likelihood and all variance component estimates between successive iterations was less than 10^−12^. All analyses were allowed to proceed until convergence, with a maximum number of iterations set according to BLUPF90 default settings.

The heritability values of clutch length and hen-day egg production under THI values were estimated by Ravagnolo and Misztal [35] with the following equation:

$$h^{2}=\frac{\sigma_{a0}^{2}+\sigma_{a1}^{2}+{2\sigma }_{a01}}{\sigma_{a0}^{2}+\sigma_{a1}^{2}+{2\sigma }_{a01}+\sigma_{p0}^{2}+\sigma_{p1}^{2}+{2\sigma }_{p01}{+\sigma }_{e}^{2}}$$

Genetic correlations $\left(r_{g}\right)$ and permanent environmental effects $\left(r_{p}\right)$ between traits (clutch length and hen-day egg production) and heat-stress effects were calculated. These values refer to the genetic components of two or more traits. They measure the extent to which the same genes or sets of genes influence multiple traits simultaneously. The equations were calculated as follows:

$$r_{g}=\frac{{COV\sigma }_{a0,a1}}{\sqrt{\sigma_{a0}^{2}\times \sigma_{a1}^{2}}}\;{,r}_{p}=\frac{{COV\sigma }_{p0,p1}}{\sqrt{\sigma_{p0}^{2}\times \sigma_{p1}^{2}}}$$

The individual estimated breeding value (EBV) of each trait according to THI values (72–82) were as follows: ${EBV}_{i({THI}_{k})}={\hat{a}}_{0}+{\hat{a}}_{1}$. The genetic trends per generation were calculated as follows: $∆G_{i}=\bar{{EBV}_{i}}-\bar{{EBV}_{i-1}}$, where *i* is generation.

## 3. Results

### 3.1. Clutch Length and Hen-Day Egg Production Performance

**Figure 1:**
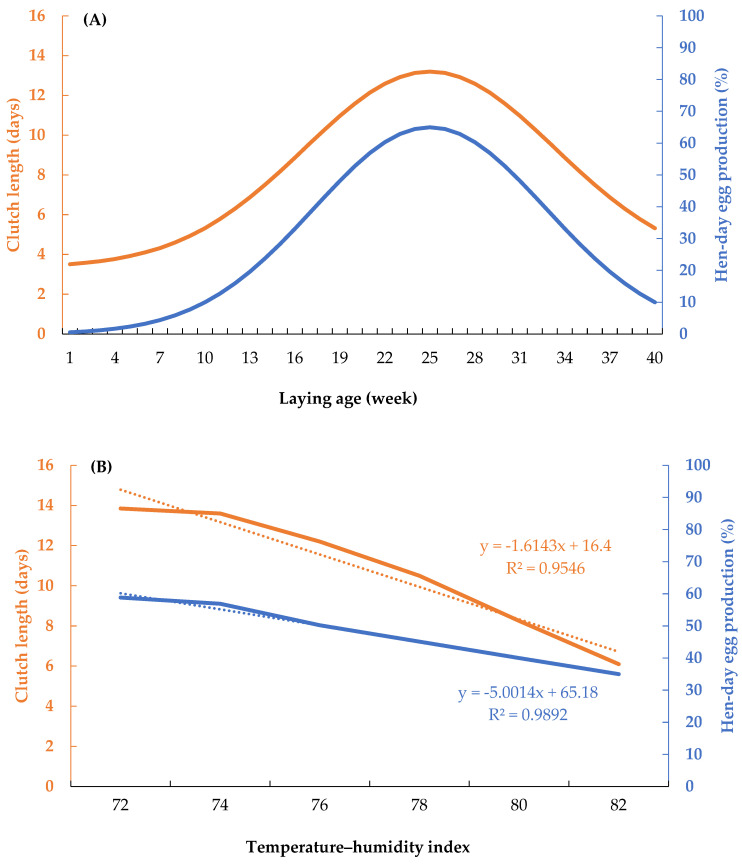
(**A**) The relationship between clutch length (orange line) and hen-day egg production (blue line) throughout the laying period and (**B**) the relationship between clutch length (orange line) and hen-day egg production (blue line) and regression lines (dotted lines) across temperature–humidity index values in Thai native chickens.

Clutch length and hen-day egg production across the laying period and under different temperature–humidity index (THI) conditions are presented in Figure 1. As shown in Figure 1A, both traits followed similar temporal patterns throughout the laying cycle. During the early laying phase (1–18 wk), clutch length increased from 3.51 eggs per clutch to 10.29 eggs per clutch. Over the same period, hen-day egg production increased from 0.53% to 43.21%. Between 19 and 26 wk of age, clutch length ranged from 10.97 to 13.13 eggs per sequence, while hen-day egg production ranged from 48.15 to 64.46%. From 27 to 40 wk of age, clutch length decreased avearge 9.21 eggs per clutch, and hen-day egg production ranged from 62.87 to 9.97%. Linear regression analyses were used to describe the relationships between the temperature–humidity index (THI) and clutch length and hen-day egg production (Figure 1B). Clutch length declined linearly with an increasing THI according to the equation used—clutch length = −1.6143 × THI + 16.40—explaining 95.46% of the variation (R^2^ = 0.9546). Similarly, hen-day egg production decreased linearly with THI, following the equation hen-day egg production = −5.0014 × THI + 65.18, with THI accounting for 98.92% of the observed variation (R^2^ = 0.9892).

### 3.2. The Effect of the THI-Determined Onset of Heat Stress and Genetic Parameters on Clutch Length and Hen-Day Egg Production

Model fit statistics (−2logL, AIC, and BIC) for clutch length and hen-day egg production across temperature–humidity index (THI) values are presented in Table 1. For both traits, these statistics differed among THI classes. For clutch length, the lowest values of −2logL, AIC, and BIC were observed at THI 74, at which point all three criteria were equal to zero. Correspondingly, higher values were observed at THI classes below and above this level. A comparable pattern was observed for hen-day egg production, with the lowest −2logL, AIC, and BIC values also recorded at THI 74, and higher values were observed at other THI classes. THI 74 was chosen as the THI threshold because it corresponds to the lower bound of the observed THI range with sufficient data density, ensuring numerical stability and reliable estimation of slopes across environments. All alternative THI thresholds were compared against this baseline, and their model fit statistics represent relative changes rather than absolute values. Therefore, a value of zero does not indicate a superior fit in isolation but instead reflects its role as the reference environment against which other THI classes are evaluated.

Genetic parameter estimates for clutch length across temperature–humidity index (THI) values are summarized in Table 1. The additive genetic variance for the baseline level (Va0) remained relatively stable at THI 72–74 (approximately 24) and showed a slight decline after THI 74. In contrast, the additive genetic variance associated with the heat-stress slope (Va1) increased progressively from 0.90 at THI 72 to 2.45 at THI 82. Estimates of the covariance between Va0 and Va1 were consistently negative, ranging from −2.13 to −3.88 across THI classes. Permanent environmental variances increased with an increasing THI, with Vp0 and Vp1 rising from approximately 15–18 at lower THI levels to 25–28 at THI 80–82. Residual variance (Ve) also increased progressively across THI classes. Correspondingly, heritability estimates for clutch length declined with an increasing THI, decreasing from h^2^ = 0.489 ± 0.04 at THI 72 to h^2^ = 0.322 ± 0.03 at THI 82.

For hen-day egg production, similar patterns were observed (Table 1). The baseline additive genetic variance (Va0) decreased gradually from 121 at THI 72 to 105 at THI 82, whereas the additive genetic variance associated with the heat-stress slope (Va1) increased from 12 to 23 across the same THI range. Covariance estimates between Va0 and Va1 were negative at all THI classes, ranging from −18.20 to −27.50. Permanent environmental variances increased with the THI, with Vp0 rising from 135 to 168 and Vp1 increasing from 15 to 22. Residual variance (Ve) also increased, rising from 60 at THI 72 to 90 at THI 82. Heritability estimates for hen-day egg production declined with an increasing THI, rising from h^2^ = 0.372 ± 0.03 at THI 72 to h^2^ = 0.263 ± 0.02 at THI 82.

Genetic correlations between clutch length and heat-stress effects were negative across all temperature–humidity index (THI) levels and became increasingly negative at higher THI values, ranging from −0.458 at THI 72 to −0.541 at THI 82. Permanent environmental correlations for clutch length showed a similar pattern, ranging from −0.687 to −0.871 across THI levels. For hen-day egg production, genetic correlations with heat-stress effects were also negative across all THI classes, with estimates ranging from −0.478 to −0.560. Permanent environmental correlations for this trait ranged from −0.520 to −0.622 across THI levels.

### 3.3. Rate of Decline

**Figure 2:**
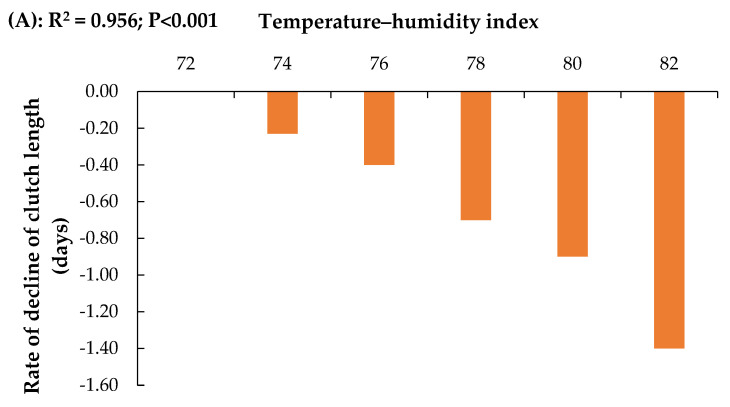

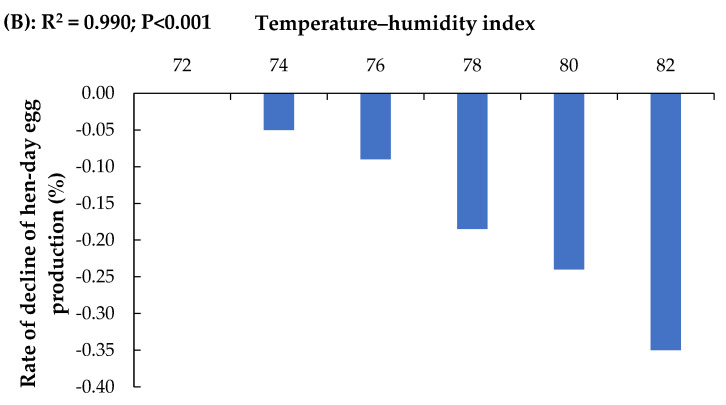
Rate of decline in (**A**) clutch length and (**B**) hen-day egg producyion in Thai native chickens at various temperature–humidity index values.

The rate of decline in clutch length and hen-day egg production across temperature–humidity index (THI) classes is presented in Figure 2. For both traits, the magnitude of decline varied across THI classes, with progressively steeper negative slopes observed at higher THI values. As shown in Figure 2A, the decline in clutch length was minimal between THI 72 and 74. An increase in the negative slope was observed at THI 76. A more pronounced decline was evident at THI 78, followed by the steepest negative slope at THI 80–82. A similar pattern was observed for hen-day egg production (Figure 2B). Declines were minimal at THI 72–74. The negative slope increased at THI 76 and became more pronounced at THI 78. The greatest decline in hen-day egg production was observed at THI 80–82.

### 3.4. Estimated Breeding Values and Genetic Gain

**Figure 3:**
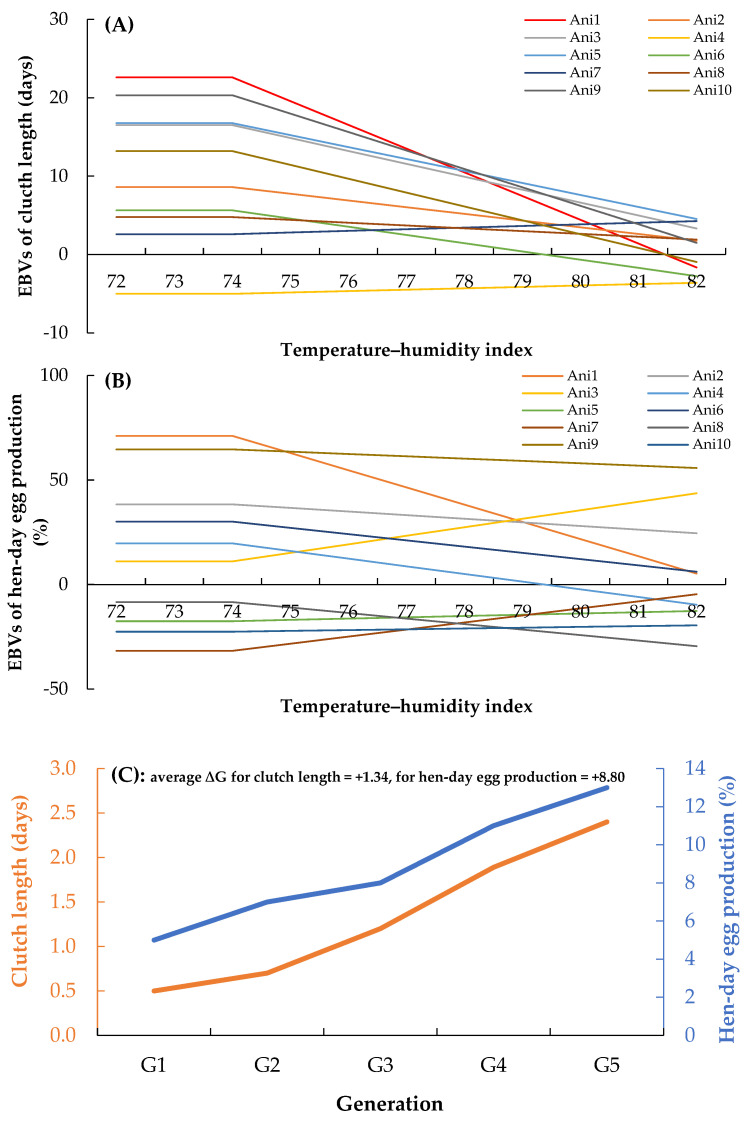
Integrated reaction-norm-based EBVs for (**A**) clutch length and (**B**) hen-day egg production under increasing THI conditions, shown for 10 randomly selected animals, and (**C**) genetic trend per generation of clutch length (orange line) and hen-day egg production (blue line) in Thai native chickens.

Estimated breeding values (EBVs) for clutch length and hen-day egg production across temperature–humidity index (THI) values, as well as genetic gain per generation in Thai native chickens, are presented in Figure 3. Figure 3A shows the reaction norms for clutch length across increasing THI values for 10 representative individuals. Under thermoneutral conditions (THI 72–74), EBVs for clutch length ranged from +1.2 to +3.8 eggs per clutch. As THI increased, EBVs declined across individuals, with differences in the magnitude of change. By THI 82, reductions ranged from 0.5–1.0 eggs for some individuals to 2.0–2.8 eggs for others. Variation in the slopes of the reaction norms was observed among genotypes across the THI gradient. Figure 3B presents the reaction norms for hen-day egg production. At thermoneutral THI values, EBVs ranged from +4% to +9% relative to the population mean. With an increasing THI, EBVs decreased noteably among individuals, although the extent of decline differed. At THI 82, reductions ranged from 3–5 percentage points for some individuals to 10–14 percentage points for others. Figure 3C illustrates the genetic trends for both traits across generations. For clutch length, the genetic trend increased from +0.5 eggs per clutch in Generation 1 to +2.4 eggs per clutch in Generation 5, corresponding to an average genetic trend of approximately +1.34 eggs per generation. For hen-day egg production, the genetic trend increased from +5.0% in Generation 1 to +13.0% in Generation 5, resulting in a genetic trend of approximately +8.8 percentage points per generation.

## 4. Discussion

Understanding how heat stress influences the genetic architecture and interrelationships of reproductive traits is essential for improving laying performance among chickens raised under tropical environments. The patterns observed between clutch length and hen-day egg production across the laying cycle reflect the close biological linkage between ovulatory rhythm and sustained egg output in laying hens [12,36]. Clutch length is widely recognized as a key determinant of egg production efficiency, as longer uninterrupted oviposition sequences indicate stable follicular recruitment, timely ovulation, and effective coordination of endocrine signals regulating the hypothalamic–pituitary–gonadal axis [37,38]. The coordinated changes in these traits across the laying period are therefore consistent with age-related shifts in ovarian activity, follicle hierarchy maintenance, and reproductive endocrine function reported in previous studies [39,40]. The observed sensitivity of both traits to increasing temperature–humidity index (THI) values aligns with well-documented effects of heat stress on reproductive performance in poultry. Elevated ambient temperatures and humidity are known to disrupt ovarian steroidogenesis, impair follicular development, and alter oviposition timing [41,42,43]. Although most evidence derives from commercial or non-native poultry populations, these studies suggest that comparable physiological pathways may also operate in native chickens, albeit with population-specific differences in genetic regulation.

Heat stress has also been shown to suppress feed intake and induce negative energy balance, further constraining ovarian function and egg formation processes [44,45]. The consistent decline in both clutch length and hen-day egg production under higher THI conditions observed in this study supports the view that reproductive traits governed by ovulatory regularity are particularly vulnerable to thermal stress. From a production perspective, these findings emphasize the importance of maintaining thermoneutral conditions during critical phases of the laying cycle to sustain reproductive efficiency. Moreover, the concurrent responses of clutch length and hen-day egg production suggest that clutch-based traits may serve as sensitive indicators of environmental stress in laying hens, complementing conventional egg production metrics. Similar conclusions have been reported in regard to commercial and native chicken populations, where clutch characteristics were proposed to be integrative traits reflecting both genetic potential and environmental adaptability [46]. Taken together, these results reinforce the relevance of incorporating environmental stress indicators, such as THI, into performance evaluation frameworks for laying hens, particularly under tropical and subtropical production systems.

Moreover, the identification of a specific THI range associated with optimal model performance suggests that thermal conditions around the thermoneutral zone represent a critical reference point for describing baseline genetic expression of reproductive traits. Similar threshold-type responses have been reported in laying hens and other poultry populations, where performance remains relatively stable until a critical THI is exceeded, after which reproductive efficiency declines rapidly [18,19,47]. The observed shift in genetic variance components across THI classes indicates that heat stress does not merely reduce overall performance but also modifies the relative contribution of genetic and environmental effects. The relative stability of baseline additive genetic variance at lower THI values, coupled with its reduction under elevated THI values, suggests that genetic differences expressed under thermoneutral conditions become increasingly masked as thermal stress intensifies. In contrast, the progressive increase in genetic variance associated with heat-stress sensitivity reflects increasing heterogeneity among genotypes in their response to heat load. This pattern is consistent with reaction-norm theory, whereby stressful environments amplify between-animal differences in environmental sensitivity rather than baseline performance [48]. As a consequence, heritability estimates declined across THI classes for both traits, reflecting a reduced proportion of phenotypic variance attributable to additive genetic effects under stressful conditions. Similar reductions in heritability under heat stress have been reported for egg production and fertility traits in commercial and native chicken populations [13,19,49]. Reduced heritability of egg traits under elevated THI values indicates that environmental variation increasingly masks genetic differences, implying that selection based solely on phenotypic performance becomes less efficient and that breeding programs may benefit from incorporating environmental sensitivity or heat-tolerance indicators into selection criteria. The increasingly negative genetic and permanent environmental correlations between baseline performance and heat-stress effects provide further evidence of genotype-by-environment interaction across the thermal gradient. These correlations indicate that individual rankings may change across THI levels, particularly under severe heat stress, reducing the predictive accuracy of breeding values estimated under thermoneutral conditions [19,50,51].

Reaction-norm-based genetic evaluation therefore offers a more robust framework for identifying animals that combine acceptable productivity with reduced environmental sensitivity [52]. Collectively, these findings are particularly relevant in the context of increasing temperature variability, which is expected to challenge the sustainability of poultry production in tropical regions [44,53,54]. The observed modification of genetic architecture for clutch length and hen-day egg production across increasing thermal loads has direct implications for breeding strategies for Thai native chickens. Accordingly, selection strategies that emphasize thermal robustness alongside optimal productivity may enhance the stability of egg production, improve animal welfare, and increase resilience to climatic variability. The progressive increase in the rate of decline in clutch length and hen-day egg production with a rising temperature–humidity index (THI) reflects the growing physiological challenge heat stress imposes on reproductive function. The nonlinear nature of this response suggests that Thai native chickens are able to maintain reproductive stability under mild thermal conditions but exhibit increasing sensitivity as environmental heat load intensifies. This pattern is consistent with previous reports indicating that indigenous chicken breeds possess the capacity to adapt to hot climates, although this capacity is finite and declines under sustained or severe heat stress [13,19]. Native chickens are generally characterized by enhanced thermoregulatory efficiency—including lower metabolic heat production and improved heat dissipation—which likely contributes to the observed resilience at lower THI levels [55]. However, as thermal stress increases, disruption of endocrine regulation, ovarian follicular development, and energy partitioning may collectively constrain ovulatory continuity and egg production [16,41]. The parallel decline patterns observed for clutch length and hen-day egg production support the notion that these traits are governed by shared physiological and regulatory mechanisms under heat stress. From a breeding perspective, the accelerated deterioration of reproductive traits at higher THI values highlights the importance of considering environmental sensitivity in genetic improvement programs.

The reaction-norm patterns observed for clutch length and hen-day egg production across increasing THI values provide clear evidence that these reproductive traits are influenced by genotype-by-environment (G×E) interactions. Differences in reaction-norm slopes among individuals indicate that genotypes respond heterogeneously to thermal stress, a pattern that has been widely reported for reproductive and production traits in poultry exposed to high ambient temperatures [56,57]. Such variability suggests that selection based solely on performance under thermoneutral conditions may not fully capture genetic differences relevant to performance under heat stress. The presence of genotypes with flatter reaction norms across the THI gradient suggests the existence of genetic variation associated with thermal robustness. Previous studies have demonstrated that birds selected for egg production in optimal environments often show greater sensitivity to heat stress, whereas indigenous or locally adapted populations tend to exhibit improved performance stability under thermal challenges [55,58,59]. The current findings are consistent with this concept and highlight the potential value of Thai native chickens as a genetic resource for heat-resilient poultry production systems. The observed divergence in reaction norms between individuals for hen-day egg production further emphasizes that heat stress does not uniformly affect all genotypes. The variation in the magnitude of decline across THI levels likely reflects underlying differences in physiological heat tolerance, including thermoregulation efficiency, endocrine responsiveness, and oxidative stress resilience [16]. From a breeding perspective, such heterogeneity reinforces the importance of incorporating environmental descriptors, such as the THI, into genetic evaluation models to better characterize performance across diverse production conditions. The positive genetic trends observed across generations indicate that sustained genetic improvement in reproductive traits is achievable despite exposure to heat stress. This aligns with previous reports showing that genomic or reaction-norm-based selection strategies can maintain or enhance genetic gain while accounting for environmental sensitivity [54]. Importantly, selection responses observed across generations suggest that incorporating heat-stress information into breeding objectives does not necessarily compromise overall genetic progress but may instead promote the accumulation of favorable alleles associated with both productivity and environmental adaptability [60,61]. Although genetic improvement is the most sustainable long-term strategy, cooling interventions remain necessary, particularly when birds are exposed to THI values > 82, which are associated with marked declines in performance. Cooling strategies such as evaporative misting, enhanced ventilation, provision of cool drinking water, and adjustment of feeding schedules to cooler periods can reduce heat load and support thermoregulation in chickens. Several limitations of the present study should be acknowledged. First, the use of a cage housing system may have limited the direct extrapolation of the results to alternative production systems, such as floor or free-range housing, where birds experience greater environmental heterogeneity and behavioral freedom. However, cage housing was intentionally selected to minimize uncontrolled environmental and management variation among individuals, thereby improving the precision of genetic parameter estimation and genotype–environment interaction analysis. Second, thermal load was quantified using the temperature–humidity index (THI), which represents a simplified indicator of heat stress and does not explicitly account for additional factors such as air velocity, radiant heat, or individual behavioral responses. Despite this limitation, the THI remains a widely used and practical metric in poultry genetic and reaction-norm studies, allowing meaningful comparison with previous research. Future studies integrating alternative housing systems and more comprehensive heat load indices may further refine genetic evaluation of reproductive traits in variable thermal environments.

## 5. Conclusions

This study evaluated genetic parameters and genotype-specific responses for clutch length and hen-day egg production in Thai native chickens across increasing temperature–humidity index (THI) conditions. The results show that heat stress alters genetic and environmental variance components and modifies the relationship between baseline performance and heat-stress sensitivity, providing evidence of genotype-by-environment interactions for reproductive traits. Reaction-norm models were effective in identifying thermal thresholds and characterizing genetic variation in environmental sensitivity, and the positive genetic trends across generations indicate that genetic improvement remains possible despite chronic heat exposure. Overall, these findings support the integration of heat-stress information and clutch-based traits into genetic evaluation systems for native chickens. Implementing THI-informed and reaction-norm-based selection strategies in breeding programs may contribute to climate-resilient genetic improvement and support sustainable poultry production under increasing climatic variability.

## Figures and Tables

**Table 1:** Variance components, genetic parameters, and model statistic criteria of clutch length and hen-day egg production among Thai native chickens at various temperature–humidity index (THI) values.

Parameters	Clutch Length						Hen-Day Egg Production					
	THI72	THI74	THI76	THI78	THI80	THI82	THI72	THI74	THI76	THI78	THI80	THI82
V_a0_	24.00	24.00	22.00	22.00	21.00	21.00	121.00	114.00	112.00	111.00	108.00	105.00
V_a1_	0.90	1.00	1.20	1.50	2.10	2.45	12.00	13.00	16.00	18.00	21.00	23.00
Cov_a0,a1_	−2.13	−2.25	−2.44	−2.78	−3.45	−3.88	−18.20	−19.50	−22.90	−24.70	−26.40	−27.50
V_p0_	15.00	16.00	18.00	22.00	25.00	28.00	135.00	145.00	148.00	155.00	160.00	168.00
V_p1_	1.89	1.99	2.22	2.86	3.33	3.99	15.00	15.00	16.00	17.00	19.00	22.00
Cov_p0,p1_	−3.66	−3.90	−4.56	−5.88	−7.21	−9.21	−23.40	−24.90	−27.80	−30.40	−33.90	−37.80
V_e_	12.00	13.00	14.00	16.00	17.50	19.40	60.00	65.00	70.00	78.00	85.00	90.00
h^2^ (±SE)	0.489 (0.04)	0.469 (0.04)	0.422 (0.03)	0.381 (0.03)	0.340 (0.03)	0.322 (0.03)	0.372 (0.03)	0.334 (0.03)	0.315 (0.03)	0.296 (0.03)	0.280 (0.03)	0.263 (0.02)
r_g_	−0.458	−0.459	−0.475	−0.484	−0.520	−0.541	−0.478	−0.507	−0.541	−0.553	−0.554	−0.560
r_p_	−0.687	−0.691	−0.721	−0.741	−0.790	−0.871	−0.520	−0.534	−0.571	−0.592	−0.615	−0.622
**Stat criteria**												
−2logL	59	0	21	83	155	276	38	0	10	75	121	178
AIC	59	0	21	83	155	276	38	0	10	75	121	178
BIC	59	0	21	83	155	276	38	0	10	75	121	178

V_a0_ = additive variance of a trait (clutch length and hen-day egg production); V_a1_ = additive variance of heat-stress effects; Cov_a0a1_ = covariance between the additive variance of a trait and heat-stress effects; V_p0_ = permanent environmental variance of a trait; V_p1_ = permanent environmental variance of heat-stress effects; Cov_p0p1_ = covariance between the permanent environmental variance of a trait and heat-stress effects; V_e_ = residual variance; h^2^ = heritability; r_g_ = genetic correlation between a trait and heat-stress effects; r_p_ = permanent environmental correlations between a trait and heat-stress effects; −2logL = minus twice the logarithm of the likelihood; AIC = Akaike’s information criterion; BIC = Bayesian information criterion.

## Data Availability

Additional data are available upon request from the corresponding author.
